# Bovine Viral Diarrhea Virus and Vaccine Protection Strategies

**DOI:** 10.3390/vetsci13020180

**Published:** 2026-02-11

**Authors:** Xinyao Hu, Jing Huang, Yafei Cai, Wei Zhang, Yun Cheng

**Affiliations:** 1 College of Animal Science and Technology, Nanjing Agricultural University, Nanjing 210095, China; 15123426@stu.njau.edu.cn (X.H.); 2022105082@stu.njau.edu.cn (J.H.); ycai@njau.edu.cn (Y.C.); 2 Jiangsu Cancer Hospital, Jiangsu Institute of Cancer Research, The Affiliated Cancer Hospital of Nanjing Medical University, Nanjing 210009, China

**Keywords:** bovine viral diarrhea virus, persistently infected animals, pathogenesis, vaccine, artificial intelligence

## Abstract

Bovine viral diarrhea virus (BVDV) is a critical pathogen affecting the global cattle industry, causing severe economic losses primarily through persistent infection, immunosuppression, and reproductive failure. The virus exhibits substantial genetic diversity, with marked geographic variation in circulating subtypes, which complicates effective disease control. BVDV evades host immune responses by suppressing type I interferon signaling, impairing neutrophil function, and reprogramming host cellular metabolism, ultimately leading to the generation of persistently infected (PI) animals that serve as the principal reservoir for viral transmission. Current prevention and control strategies rely mainly on the identification and elimination of PI animals in combination with vaccination. However, conventional vaccines, including inactivated vaccines (IVs) and modified live vaccines (MLVs), have notable limitations, such as suboptimal subtype matching, interference by maternal antibodies, and safety concerns associated with MLV use in pregnant cattle. Emerging vaccine platforms, including mRNA vaccines, subunit vaccines, and multi-epitope vaccines, offer promising alternatives owing to their improved safety profiles, rapid design and production, and potential to elicit broad and robust immune responses. Future BVDV vaccine development should integrate artificial intelligence-driven design strategies with high-throughput sequencing and molecular epidemiological surveillance to enable the rational development of multivalent and multi-epitope vaccines. In addition, coordinated implementation of strain monitoring, PI animal clearance, and enhanced biosecurity practices will be essential for establishing a comprehensive and sustainable BVDV prevention and control framework.

## Simple Summary

Bovine viral diarrhea virus (BVDV) is a major infectious agent threatening the global cattle industry. It causes persistent infections and a wide spectrum of clinical manifestations, leading to substantial economic losses. The extensive genetic diversity of BVDV and the regional heterogeneity of circulating strains pose significant challenges for effective vaccine-based prevention and control. This review summarizes the transmission characteristics, pathogenic mechanisms, and current vaccine strategies against BVDV. We highlight the limitations of conventional vaccines, particularly with respect to safety concerns and incomplete protective coverage, and discuss recent advances in novel vaccine platforms, including mRNA- and subunit-based vaccines. Furthermore, we emphasize that future BVDV vaccine development should integrate artificial intelligence-assisted design and molecular surveillance to achieve broader and more precise protection. Combined with systematic identification and removal of infected animals and strengthened biosecurity measures, these approaches may enable the establishment of an efficient and integrated BVDV prevention and control system, ultimately improving herd health and livestock productivity with substantial societal and economic benefits.

## 1. Introduction

### 1.1. Characteristics and Classification of BVDV

Bovine viral diarrhea virus (BVDV) is a member of the genus *Pestivirus* within the family *Flaviviridae* and is the etiological agent of bovine viral diarrhea (BVD), one of the most economically important infectious diseases affecting cattle worldwide. BVDV is an enveloped, positive-sense, single-stranded RNA virus with a genome of approximately 12.3 kb [1]. Based on antigenic and genetic diversity, BVDV is classified into several genotypes. These include BVDV-1 (designated Pestivirus A by the International Committee on Taxonomy of Viruses, ICTV), which comprises 21 subtypes (1a–1u); BVDV-2 (Pestivirus B), which includes four subtypes (2a–2d); and BVDV-3 (Pestivirus H), also consisting of four subtypes (3a–3d) [1,2]. Among these, BVDV-1 is the most widely distributed globally. Subtypes 1a and 1m predominate in Asia, subtype 1d is most prevalent in Europe, and all three subtypes are commonly detected in the Americas [3,4,5,6].

The control of BVDV is particularly challenging because the virus can be transmitted both vertically and horizontally, with vertical transmission leading to the generation of persistently infected (PI) animals. This phenomenon results from the virus’s immune evasion strategy. When a fetus is infected with BVDV during early gestation, its developing immune system recognizes the virus as self and fails to mount an effective immune response. Hence, the animal is born immunotolerant to BVDV and remains persistently infected, continuously shedding the virus throughout its life [7]. Based on cytopathogenicity in cell culture and biological characteristics, BVDV is classified into two biotypes: cytopathic (cp) and non-cytopathic (ncp). PI animals exclusively harbor the ncp biotype, which serves as the primary reservoir for viral persistence and transmission while maintaining lifelong immune tolerance. Within PI animals, continuous replication of ncp BVDV provides abundant opportunities for random mutations as well as viral or cellular RNA recombination. These processes may give rise to a novel virus that is antigenically homologous to the parental strain but exhibits the cp biotype. The emergence of cp BVDV is typically associated with genomic insertions, duplications, or rearrangements, which alter polyprotein processing, profoundly affect virus–host interactions, and often result in enhanced viral replication and pronounced cytopathic effects [8]. In addition to vertical transmission, PI animals represent a major source of horizontal spread, as they continuously excrete high titers of infectious virus in nasal and ocular secretions, urine, semen, milk, and feces [9].

Persistent BVDV infection induces immunosuppression, predisposing animals to co-infections with multiple pathogens and leading to severe immune dysfunction. Clinical manifestations may include oral mucosal lesions, mucosal disease, enteric disorders, and respiratory disease. Masebo et al. identified BVDV as a critical initiating factor in bovine respiratory disease (BRD) syndrome, demonstrating that BVDV-induced immunosuppression markedly increases herd susceptibility to secondary pathogens, such as Mycoplasma bovis, thereby elevating BRD incidence. BRD is associated with reduced weight gain, decreased feed efficiency, increased mortality and culling rates, and carcass downgrading or condemnation due to pulmonary lesions at slaughter. Effective control of BVDV is therefore essential for reducing BRD susceptibility, lowering antimicrobial use, improving animal welfare, and enhancing the overall economic efficiency of beef production systems [10,11]. Clinically, BVDV infection can result in acute disease characterized by fever, leukopenia, and diarrhea, or lead to lifelong persistent infection. Both outcomes are associated with reproductive disorders and an increased risk of secondary infections [12,13].

The economic impact of BVDV is substantial, with annual losses to the cattle industry estimated at 1.5–2.5 billion US dollars, corresponding to approximately 0.5–687.8 dollars per cow, depending on production system and disease prevalence [1,14]. Arnaiz et al. demonstrated that BVDV infection significantly compromises reproductive performance in dairy cows and that timely vaccination can effectively mitigate these adverse reproductive outcomes, suggesting the critical importance of vaccination in BVDV prevention and control strategies [15].

### 1.2. Global Distribution and Epidemiological Status of BVDV

**Figure 1:**
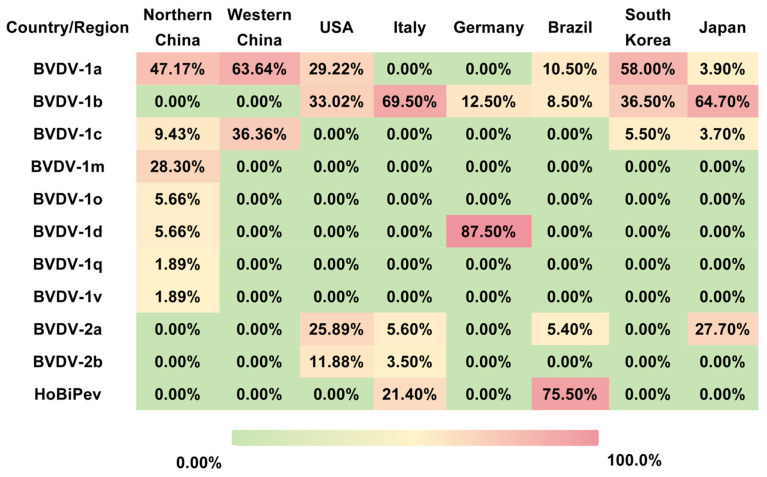
Geographical distribution of BVDV subtypes in selected countries and regions. The table summarizes the prevalence (%) of major BVDV subtypes in Northern China, Western China, the United States, Italy, Germany, Brazil, South Korea, and Japan. The data illustrate pronounced regional differences in subtype dominance, such as BVDV-1a in China and South Korea, BVDV-1b in Italy and Japan, BVDV-1d in Germany, and HoBi-like pestivirus (BVDV-3) in Brazil. These disparities highlight the necessity of regionally tailored surveillance programs and vaccine design strategies for effective BVDV control.

BVDV is distributed worldwide; however, the predominant genotypes and subtypes vary considerably among countries and regions (Figure 1). BVDV-1 predominates in countries such as China, Germany, and Sweden, whereas BVDV-3 (HoBi-like pestivirus, HoBiPeV) is most prevalent in Brazil. In the United States, both BVDV-1 and BVDV-2 are widely circulating, while Italy reports the co-circulation of all three virus species, BVDV-1, BVDV-2, and BVDV-3, with substantial genetic diversity [16].

In China, several studies have documented extensive genetic heterogeneity and distinct regional distribution patterns of BVDV. For example, three BVDV strains isolated from diarrheic calves were identified by whole-genome sequencing as BVDV-1a, BVDV-1m, and a novel subtype, BVDV-1v. Experimental infection of calves demonstrated marked differences in pathogenicity, with BVDV-1m exhibiting high virulence, whereas BVDV-1a and BVDV-1v showed moderate pathogenicity [17]. Large-scale epidemiological investigations further revealed pronounced geographic clustering of BVDV subtypes. In western China (including Shaanxi, Ningxia, Xinjiang, and Tibet), the animal-level prevalence of BVDV was approximately 7.2%, with herd-level infection rates reaching up to 82.4%. In these regions, BVDV-1a and BVDV-1c were the dominant subtypes [18]. In contrast, northern China is characterized by the predominance of BVDV-1a and BVDV-1m. A survey covering 13 northern provinces reported an overall BVDV positivity rate of 6.05%, with subtype frequencies of 47.17% for BVDV-1a, 28.30% for BVDV-1m, and 9.43% for BVDV-1c. Notably, no BVDV-2 or BVDV-3 strains have been reported in these regions to date [19]. However, the highly pathogenic BVDV-1b strain BJ-11 was identified in Beijing, suggesting localized evolution and the emergence of virulent variants within northern China. Collectively, these findings indicate that BVDV-1 is the dominant genotype in China, with subtypes 1a and 1c prevailing in western and northern regions, alongside sporadic emergence of highly virulent subtype 1b strains [20]. Geographic differences in subtype distribution likely reflect variations in production systems, animal movement, and vaccination practices, highlighting the importance of region-specific strain matching in vaccine development.

Outside China, phylogenetic and phylogeographic studies have provided insights into the global spread and evolution of BVDV. In the United States, BVDV-1 and BVDV-2 are estimated to have diverged in the 1670s and 1890s, respectively. Subtypes BVDV-1a, BVDV-1b, BVDV-2a, and BVDV-2b are currently the most prevalent and widely distributed. Phylogeographic analyses further identify the United States as a major dissemination hub for both BVDV-1 and BVDV-2, with frequent interregional transmission events contributing to viral spread [21]. HoBiPeV is believed to have originated in Brazil during the 1980s, and Brazil remains the principal endemic country for this virus in South America. The northeastern region of Brazil represents a hotspot of HoBiPeV circulation, where HoBiPeV-a is the dominant endemic subtype [22,23]. Italy exhibits one of the highest levels of BVDV genetic diversity worldwide, with BVDV-1, BVDV-2, and HoBiPeV co-circulating. Among these, BVDV-1 is the most prevalent, with at least 15 identified subtypes, particularly 1b and 1e, distributed throughout the country, whereas BVDV-2 and HoBiPeV are detected sporadically [24]. The distribution of BVDV subtypes exhibits distinct geographic clustering patterns (Figure 1).

Several European countries have implemented successful national eradication programs that have profoundly altered the epidemiological landscape of BVDV. In Germany, a mandatory eradication program initiated in 2011, centered on systematic detection and removal of PI animals, led to a dramatic reduction in PI prevalence. By 2024, the PI detection rate had declined to 0.001%, with only 46 PI calves identified nationwide. Concurrently, viral diversity decreased markedly, shifting from multiple circulating subtypes to a predominance of BVDV-1d, with subtype 1b detected only in the state of Schleswig-Holstein, indicating substantial homogenization of virus transmission [25]. Similarly, Switzerland launched a mandatory national BVDV eradication program in 2008. The program initially relied on herd-wide virus screening and continuous testing of newborn calves to identify and eliminate PI animals, followed by a transition to serological surveillance and a complete ban on vaccination. As a result, approximately 99.5% of Swiss farms were free of BVDV by the end of 2020. Nevertheless, the identification and removal of the remaining PI animals remain the most challenging phase of the eradication effort [26].

China, Germany, and Sweden all report BVDV-1 as the predominant genotype. However, Germany and Switzerland have achieved exceptionally low prevalence and marked subtype homogenization through the implementation of mandatory national eradication programs. In contrast, China remains in a phase characterized by extensive viral diversity, with BVDV prevention and control relying largely on localized vaccination strategies and without a nationwide system for the systematic detection and elimination of persistently infected animals [25]. The German experience demonstrates that comprehensive surveillance, compulsory removal of PI animals, stringent animal movement control, and sustained regulatory oversight are central pillars of successful BVDV eradication. These measures provide valuable insights not only for China but also for other regions seeking to establish effective BVDV control frameworks. For China, the principal challenges extend beyond the heterogeneity of cattle production systems and uneven surveillance capacity. A critical technical bottleneck lies in the development of vaccines that are precisely matched to locally prevalent BVDV subtypes, which remains one of the core tools for domestic disease prevention and control. Although such region-specific approaches are essential for constructing an effective national BVDV control system, their applicability to a unified global vaccine strategy is currently limited. Accordingly, these country-specific considerations are not included in the central analytical focus of this review.

Importantly, the geographic distribution of BVDV is not static but undergoes continuous dynamic change under the influence of multiple interacting factors. Among these, climate change and the globalization of livestock trade appear to exert particularly strong and synergistic effects on the redistribution of BVDV. Although direct evidence linking climate variables and global trade to BVDV dissemination remains limited, their impact can be inferred from studies on bovine respiratory disease (BRD). Long-distance transportation associated with international trade represents a major conduit for pathogen spread and co-infection, substantially increasing both the prevalence and diversity of respiratory pathogens in transported cattle. Concurrently, climatic stressors, including temperature extremes and fluctuations in humidity, can impair host immune function and create conditions favorable for viral transmission and persistence [27]. Together, these factors may facilitate the introduction of BVDV into previously unaffected regions and complicate control efforts in endemic areas by reshaping transmission dynamics and host–pathogen interactions. Therefore, assessments of the current epidemiological landscape of BVDV should be complemented by future investigations into the drivers of viral spread to inform more comprehensive and adaptive control strategies.

### 1.3. Challenges in BVDV Vaccine Prevention and Control

Given the pronounced regional heterogeneity in BVDV subtype distribution, vaccine development and implementation face substantial challenges. Despite considerable advances in understanding BVDV biology and epidemiology, existing prevention and control strategies, particularly vaccination, remain constrained by several critical limitations. Foremost among these is the extensive genetic and antigenic diversity of BVDV. Most commercially available vaccines are based on historically prevalent subtypes, such as BVDV-1a and BVDV-1b, and often provide suboptimal cross-protection against newly emerging or regionally dominant subtypes, including BVDV-1d and BVDV-1e [28]. The limited heterologous protection afforded by current vaccines has been demonstrated experimentally. For example, even when dams were repeatedly vaccinated prior to conception with an inactivated virus (IV) vaccine derived from BVDV-1a, their offspring were subsequently infected with BVDV-1d and developed persistent infection [7]. Moreover, accumulating evidence suggests that widespread and prolonged vaccine use may exert selective pressure on viral evolution, potentially contributing to shifts in subtype prevalence and increased epidemiological diversity. Beyond viral genetics, ecological and epidemiological factors further complicate BVDV control. Wildlife and non-bovine domestic species, such as deer and alpacas, can serve as alternative hosts and viral reservoirs, facilitating viral maintenance, cross-species transmission, and genetic diversification [9].

Collectively, these factors highlight why BVDV remains difficult to control using vaccination alone. Furthermore, many existing reviews provide limited coverage of newly emerging BVDV subtypes and do not fully incorporate recent technological advances, including artificial intelligence (AI)-driven approaches. While these emerging technologies enable precise and rapid surveillance of emerging BVDV subtypes, current review articles have paid insufficient attention to this critical aspect. Our review thus focuses on elaborating the future development trends and prospects in this domain. Future progress in BVDV prevention will likely depend on optimizing vaccine design through dynamic updating of vaccine strains based on real-time epidemiological surveillance, as well as the adoption of innovative platforms capable of eliciting broader and more durable cross-protective immune responses. Importantly, vaccination must be integrated with systematic detection and removal of PI animals and reinforced by comprehensive biosecurity measures. Such a coordinated strategy is essential for establishing a sustainable, multidimensional framework for effective BVDV prevention and control.

## 2. Methods

A comprehensive literature review of BVDV vaccines was conducted following the methodological framework described in a previous review of BVDV immune responses [1]. The objective was to critically assess the current status and limitations of conventional BVDV vaccines and to provide an in-depth evaluation of emerging vaccine strategies, with particular emphasis on their feasibility and potential advantages. This review was guided by three central questions: (i) how BVDV interacts with the host immune system; (ii) the strengths and weaknesses of existing conventional and novel vaccine platforms; and (iii) how emerging technologies can be integrated into future BVDV vaccine development.

The scope of the review was restricted to peer-reviewed English-language publications published between 2020 and 2025 that addressed BVDV subgenotypes, global distribution and disease burden, host immune responses, vaccine development, and novel technologies relevant to vaccine design. Two complementary electronic databases, PubMed and Web of Science, were systematically searched. In parallel, reference lists of all included articles were manually screened to identify key studies that may not have been captured in the initial database searches.

The search strategy employed the following keywords and combinations thereof: “BVD,” “BVDV,” “bovine viral diarrhea virus,” “vaccin*,” “vaccines,” “conventional vaccines,” “mRNA vaccine*,” “subunit vaccines,” “cell-mediated immunity,” “adaptive immunity,” “humoral immunity,” “T lymphocytes,” “B lymphocytes,” “innate immune cells,” “innate immune response,” “maternal antibody,” and “immune*.” Articles were selected based on the depth and rigor of their analysis of the current epidemiological and biological status of BVDV, the accuracy with which they characterized interactions between BVDV and the host immune system, and the degree of innovation proposed for improving existing BVDV vaccine strategies.

**Figure 2:**
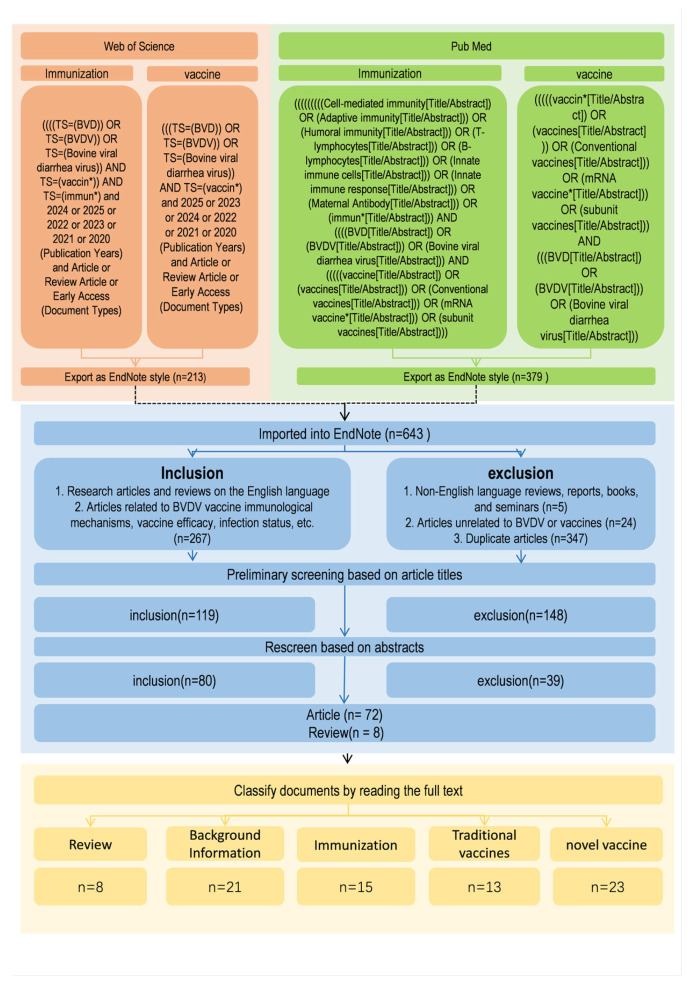
Flowchart of the literature screening and selection process for the review of BVDV vaccines. The diagram outlines the sequential steps used to identify, screen, and select publications for inclusion. Searches were conducted in PubMed and Web of Science using predefined keywords related to BVDV, vaccines, and immune responses. Records were screened by title, abstract, and full-text review according to established inclusion and exclusion criteria. Following duplicate removal and exclusion of irrelevant studies, 80 articles (72 original research articles and 8 reviews) were retained for detailed qualitative analysis and thematic categorization.

An initial screening of retrieved records was conducted based on titles and abstracts using predefined inclusion and exclusion criteria. Studies that met these criteria were subsequently subjected to full-text evaluation to confirm their relevance, data adequacy, and conceptual contribution to the field. A formal, standardized quality assessment tool was not applied to quantitatively score individual studies. Database searches retrieved 263 records related to “BVDV vaccine” and 116 related to “BVDV immunization” from PubMed, and 213 and 51 corresponding records from the Web of Science database, respectively. After removal of duplicates and exclusion of reviews, reports, books, conference proceedings, non-full-text articles, non-English publications, and studies not directly related to BVDV vaccines or immunization, a total of 80 articles were included in the final analysis, comprising 72 original research articles and 8 review articles (Figure 2).

To ensure consistency and reproducibility of the literature retrieval process, the review was restricted to English-language publications. This decision was based on the relatively standardized peer-review processes commonly employed by international English-language journals, which provide a degree of quality assurance for the included studies. Nevertheless, this language restriction may have resulted in the exclusion of relevant research published in other languages, particularly from regions such as South America and Asia, and should therefore be considered a limitation of this review.

## 3. Pathogenic Mechanisms of BVDV

**Figure 3:**
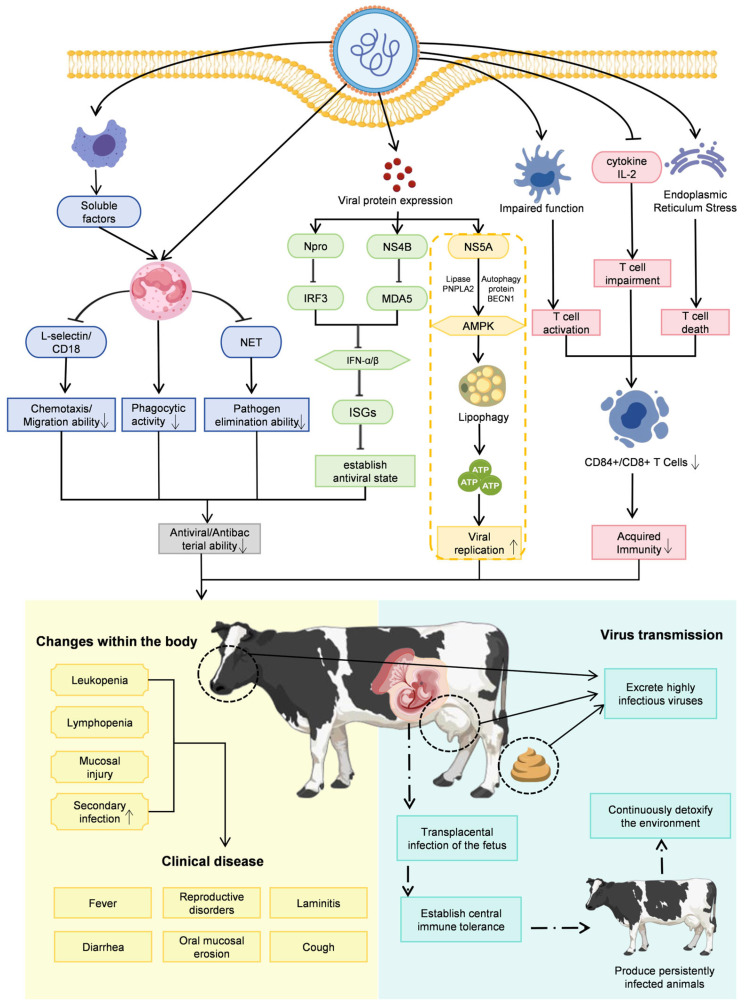
Schematic overview of the key pathogenic mechanisms of BVDV. BVDV causes disease, persistence, and herd-level transmission through a combination of five interrelated mechanisms. Solid line arrows indicate promotion; bar-ended lines indicate inhibitory effects; broken line arrows indicate sequential progression; upward or down-ward arrows inside boxes indicate increase or decrease. First, innate immune suppression is mediated by nonstructural proteins, with N^pro^ promoting IRF3 degradation and NS4B interacting with MDA5 to inhibit type I interferon (IFN-α/β) production, preventing the establishment of an antiviral state. Second, neutrophil dysfunction arises as soluble factors from virus-infected macrophages downregulate L-selectin and CD18, impairing chemotaxis, phagocytosis, and neutrophil extracellular trap (NET) formation. Third, adaptive immune disruption results from reduced CD4^+^ and CD8^+^ T-cell counts and diminished antiviral cytokine production, contributing to systemic immunosuppression. This directly leads to increased susceptibility to secondary infections. Fourth, metabolic hijacking occurs when NS5A interacts with host autophagy protein BECN1 and lipase PNPLA2, activating AMPK and lipophagy to mobilize free fatty acids for mitochondrial β-oxidation, providing energy for viral replication. Fifth, persistent infection (PI) establishment is achieved through transplacental infection during early gestation, inducing central immune tolerance in the fetus and producing PI animals that continuously shed high viral loads, acting as the primary reservoir for herd transmission. This closely links individual pathology with population epidemiology. Together, these mechanisms lead to clinical manifestations including leukopenia, mucosal lesions, diarrhea, fever, reproductive disorders, and increased susceptibility to secondary infections.

The pathogenicity of BVDV arises from complex and multifactorial interactions between the virus and the host immune system. On the one hand, the viral envelope glycoprotein E2 is highly immunogenic and capable of eliciting robust protective immune responses, including the induction of neutralizing antibodies. On the other hand, BVDV has evolved a range of sophisticated immune evasion and immunosuppressive strategies that undermine these host defenses. These include the suppression of innate and adaptive immune responses mediated by viral nonstructural proteins, as well as the induction of immunological tolerance during fetal development, leading to the generation of PI animals. Together, these mechanisms enable long-term viral persistence, facilitate ongoing transmission, and contribute to the development of severe and diverse clinical manifestations in infected hosts (Figure 3).

The capacity of BVDV to suppress the host innate immune response represents a central determinant of its pathogenicity. This immune antagonism is primarily mediated by viral nonstructural proteins (NSPs), including N^pro^, NS2, NS3, NS4A, NS4B, NS5A, and NS5B. Among these, N^pro^ plays a pivotal role by inducing the ubiquitination and proteasomal degradation of interferon regulatory factor 3 (IRF3), thereby blocking type I interferon (IFN-α/β) induction. In parallel, NS4B interacts with the caspase activation and recruitment domains (2CARD) of the host pattern-recognition receptor MDA5, suppressing downstream antiviral signaling and further inhibiting type I interferon production. Through these coordinated mechanisms, BVDV effectively evades innate immune surveillance and establishes an early permissive environment for viral replication [6,29,30,31]. As illustrated in Figure 3, such active suppression of innate immunity serves as the key initial step facilitating the successful establishment of primary infection by viruses and the creation of a conducive environment for their subsequent replication.

Building on this initial innate immune evasion, BVDV achieves long-term persistence by profoundly manipulating the adaptive immune system. Persistent infection arises when fetal exposure to BVDV during early gestation induces central immune tolerance involving both B and T lymphocyte lineages, thereby circumventing adaptive immune recognition [8]. Because maternal antibodies are unable to cross the epitheliochorial placenta of cattle, early gestational infection allows the virus to reach the fetus unopposed. The developing fetal immune system subsequently misidentifies the replicating virus as self, fails to mount antiviral responses, and gives rise to PI animals. These PI cattle are unable to clear the virus and continuously shed large quantities of infectious virus, making them the most important reservoir for BVDV transmission. This corresponds to the core pathway of “transplacental infection-immune tolerance” depicted in Figure 3. This mechanism links individual pathology with population epidemiology, elucidating how individual PI animals become the most critical viral reservoirs and sources of infection within a population. Thus, PI animals exert long-term and widespread effects on herd health and productivity, contributing to reproductive losses such as abortion, stillbirth, and congenital malformations, as well as increased susceptibility to secondary infections throughout life [26,32].

The pathogenesis of mucosal disease provides a classic illustration of BVDV-induced immune tolerance. During fetal development, PI animals acquire central tolerance to the ncp biotype of BVDV. As a result, the adaptive immune system fails to recognize a subsequently arising cp biotype, often generated through mutation or recombination, as foreign. In the absence of effective immune control, the cp virus undergoes rapid replication and induces extensive cytolysis, particularly in mucosal tissues, resulting in severe and often fatal clinical disease. Thus, early evasion of innate immunity is a prerequisite for the establishment and maintenance of persistent infection and the downstream development of mucosal disease [7,8].

Beyond direct virus–host protein interactions, BVDV also exerts indirect immunosuppressive effects. Abdelsalam et al. demonstrated that BVDV-infected macrophages secrete soluble mediators that downregulate the expression of L-selectin and CD18 on neutrophils, thereby impairing their chemotaxis, migration, phagocytic capacity, and oxidative burst activity. Notably, cytopathic BVDV further suppresses neutrophil function by inhibiting nitric oxide production and neutrophil extracellular trap (NET) formation, resulting in profound defects in innate immune defense [33]. Simultaneously, BVDV directly modulates T-cell responses by inducing upregulation of CD25 (the interleukin-2 receptor α chain) and increasing intracellular interferon-γ expression within T-cell subsets. These alterations are associated with a reduction in circulating CD4^+^ and CD8^+^ T lymphocytes and overall lymphopenia, thereby weakening host immunity to heterologous pathogens [34]. Together with disruption of the macrophage–neutrophil axis, these immunomodulatory effects render BVDV-infected animals highly susceptible to secondary infections, which frequently manifest as severe bacterial co-infections and contribute substantially to disease severity and economic losses [35]. Although BVDV deploys multiple, layered strategies to suppress host immunity, the host immune system does mount counteracting responses, in which the viral structural glycoprotein E2 plays a pivotal role by eliciting neutralizing antibodies and protective adaptive immunity.

The structural glycoprotein E2 of BVDV is a potent stimulator of both humoral and cellular immune responses. In the humoral arm, E2 is the primary target of neutralizing antibodies due to its strong immunogenicity [36,37]. Most monoclonal antibodies directed against E2 exhibit viral neutralizing activity, primarily by recognizing conformational epitopes within structural domain A [38,39]. These epitopes are resistant to acid treatment, but their three-dimensional structure is disrupted upon reduction of disulfide bonds, indicating that the protective humoral response elicited by E2 relies on the integrity of its conformational epitopes. This stability under acidic conditions further supports the potential of E2 to stimulate effective neutralizing antibody responses during infection [40,41]. At the cellular immunity level, CD4^+^ and CD8^+^ T-cell responses targeting E2 are critical for establishing protective immunity. During viral replication, the E2 protein is processed into peptides that are presented via major histocompatibility complex (MHC) class I and class II molecules to CD8^+^ and CD4^+^ T cells, respectively, activating virus-specific T-cell responses [42,43,44,45]. The dual capacity of E2 to elicit both humoral and cellular immune responses underscores its value as a key antigen for vaccine development and as a diagnostic target.

Beyond immune modulation, BVDV replication relies heavily on hijacking host cell metabolic pathways. For instance, Nonstructural Protein 5A (NS5A) has been shown to orchestrate systematic reprogramming of host energy metabolism. NS5A interacts with autophagy core protein Beclin 1 (BECN1) and the lipase adipose triglyceride lipase (ATGL/PNPLA2), leading to a reduction in intracellular ATP levels. This energy depletion activates AMP-activated protein kinase (AMPK), which in turn triggers lipophagy. Lipophagy enhances ATGL-mediated breakdown of lipid droplets, releasing free fatty acids that are transported into mitochondria for β-oxidation, thereby generating ATP to fuel viral replication [46]. This process, termed “metabolic hijacking” in Figure 3, reveals how viruses sustain their massive replication by redistributing energy resources.

Collectively, BVDV combines immune evasion strategies with metabolic hijacking to drive disease pathogenesis. Fetal infection enables the virus to bypass the developing immune system, establishing persistent infection; these PI animals subsequently become the principal source of viral dissemination within herds, highlighting the key role of immune escape in BVDV epidemiology and pathogenesis. Simultaneously, BVDV suppresses innate immunity, impairs neutrophil function, and promotes secondary infections, while exploiting host lipid metabolism to support viral replication.

## 4. Traditional Vaccines

Insights into BVDV’s immune evasion and metabolic hijacking inform rational vaccine design. Effective control of BVDV requires an integrated strategy: strict biosecurity, identification and removal of PI animals, and vaccination. Among these, vaccination remains a cornerstone in the dairy and beef industries for reducing clinical disease and limiting viral spread. Since PI animals are the principal source of viral transmission and central to herd-level persistence, a key objective of vaccination is to elicit robust immunity that protects both adult cattle and the fetuses of pregnant cows, thereby preventing the generation of new PI animals at the source [47].

### 4.1. Inactivated Virus Vaccines

Inactivated BVDV vaccines are well-established for their ability to stimulate both humoral and cellular immune responses. For example, in a mouse model, inactivated vaccines not only promoted the production of interferon-gamma (IFN-γ) by splenocytes, which increased over time, but also induced high levels of immunoglobulin G (IgG) and its subclasses (IgG1a and IgG2a), thereby enhancing overall immunity [48,49].

Current research on inactivated vaccines focuses on optimizing inactivation methods and adjuvant formulations. One study demonstrated that hydrogen peroxide (H_2_O_2_) inactivation elicited higher levels of BVDV-specific IgG and neutralizing antibodies, as well as a more balanced Th1/Th2 cellular response, reflected in elevated IFN-γ and IL-4, with longer-lasting immunity compared to conventional formaldehyde-inactivated vaccines [3]. Regarding adjuvants, monolaurin-based formulations were shown to sustain neutralizing antibody titers for up to nine months, outperforming traditional aluminum-adjuvanted vaccines while avoiding localized adverse reactions [14]. Moreover, multivalent vaccines incorporating locally prevalent strains (e.g., BVDV-1f and BVDV-2b) and potent oil-based adjuvants such as Montanide^®^ ISA 206 elicited stronger, longer-lasting, and cross-protective humoral responses compared to aluminum-based formulations [50].

IV vaccination protocols usually comply with specific procedures established by manufacturers. The primary immunization course comprises two doses administered 2 to 4 weeks apart to induce initial immune memory. Notably, IV vaccines fail to confer lifelong protection, with a protective duration of 6 months to 1 year, thus necessitating regular boosters to sustain sufficient antibody titers [51]. Given their favorable safety profile, IV vaccines are applicable for pregnant cows, though the timing of vaccination is strictly regulated—to ensure the dam acquires robust immune protection during the critical gestational period and thereby minimize the risk of PI calf delivery as much as possible [7].

Despite their high safety profile, inactivated vaccines have limitations. Their protective efficacy is strongly dependent on antigenic matching between vaccine strains and circulating field strains. For instance, an outbreak caused by BVDV-1r occurred in a farm routinely using an inactivated vaccine based on BVDV-1a (NADL strain), resulting in a high proportion of PI calves, which are infected with BVDV during the critical window of fetal immune system development, recognize viral antigens as self-components and thus fail to mount an effective adaptive immune response against BVDV antigens in IV vaccines, highlighting the vulnerability of IV vaccines to antigenic mismatch [52,53].

In summary, while inactivated vaccines induce robust immune responses, they exhibit an excellent safety profile and are thus well-suited for vulnerable populations, including pregnant animals. Research indicates that optimizing inactivating agents and adjuvants can further enhance antibody titers, prolong the duration of immunity, and improve immune responses, thereby achieving protection that lasts from several months to a year in practice. However, the immunogenicity of this platform is highly dependent on antigenic match between vaccine and circulating strains. Significant antigenic divergence—such as when the vaccine strain is BVDV-1a, but the circulating strain is BVDV-1r—can lead to protection failure or even persistent infection in calves, even with standardized immunization protocols. Future research should focus on improving inactivation methods, optimizing adjuvants, and aligning vaccine strains with locally circulating variants to develop next-generation inactivated vaccines with enhanced immunogenicity and cross-protective efficacy.

### 4.2. Modified Live Viral (MLV) Vaccine

MLV vaccines for BVDV are highly effective in eliciting robust humoral and cellular immune responses, providing strong protection against infection. However, the immunogenicity and protective efficacy of MLV vaccines are influenced by the specific viral strain used in the formulation [42,47].

For example, Małgorzata et al. demonstrated that a single dose of a commercial MLV BVDV vaccine rapidly induced a strong humoral response, with antibody levels significantly increasing within one month, peaking at 2–3 months, and remaining elevated for at least 12 months. Importantly, no adverse effects were observed in vaccinated animals, supporting the practical application of MLV vaccines in the field [42].

The choice of vaccine strain also critically affects cross-protective immunity. Fulton et al. reported that an MLV vaccine containing BVDV-1a (Singer strain) was superior to one containing BVDV-1a (NADL strain) in generating cross-neutralizing antibodies, particularly against the predominant BVDV-1b subtype in the United States. The Singer-based vaccine enabled a higher proportion of vaccinated cattle to achieve fetal-protective antibody titers (≥128). By contrast, inactivated vaccines showed intermediate efficacy against BVDV-1b, protecting only ~20% of vaccinated animals, highlighting the advantage of MLV vaccines in inducing broad, high-level immunity against circulating subtypes [47].

Beyond homotypic protection, MLV vaccines have demonstrated cross-protective potential against heterologous strains. For instance, a multivalent MLV vaccine targeting BVDV-1 and BVDV-2 conferred approximately 80% fetal protection when cows were challenged with BVDV-3, indicating that MLV-induced immunity extends beyond neutralizing antibodies and may involve cellular immune mechanisms [54,55]. Supporting this, studies have shown that MLV vaccination effectively primes the immune system, stimulating robust cellular responses characterized by increased IFN-γ expression in CD4^+^ and CD8^+^ T cells, as well as CD335^+^ natural killer (NK) cells, along with enhanced expression of the T cell activation marker CD25 following viral restimulation [44].

MLV vaccines mimic the natural infection process by utilizing attenuated yet replication-competent viruses, a characteristic that underpins their ability to induce rapid, comprehensive, and long-lasting immune responses. A single vaccination rapidly elicits high levels of neutralizing antibodies and induces robust cellular immunity, thereby establishing broad immune memory. These immunological advantages translate directly into significant practical benefits, such as providing highly effective and long-lasting fetal protection, while also demonstrating potential for cross-protection against heterologous strains [56,57]. According to Table 1, among the currently available commercial vaccines for BVDV, the two most effective are the MLV vaccines Mucosiffa^®^ and Breed Back^®^10. The former provides longer-lasting protection, while the latter induces higher antibody titers.

Nevertheless, they have inherent limitations: MLV vaccines can establish latent infections that may reactivate under stress, hormonal changes, or immunosuppression. Vaccination of pregnant cows carries a risk of fetal infection, potentially causing early embryonic death or abortion. Both MLV and inactivated vaccines also face reduced effectiveness in the presence of maternally derived antibodies (MatAb), which can neutralize the vaccine virus and blunt the initial immune response [58].

Indeed, studies have shown that in young calves with high levels of MatAb, neither MLV nor inactivated vaccines elicited significant antibody responses following initial vaccination. Moreover, antibody titers often declined post-vaccination, highlighting the critical challenge posed by maternal immunity in undermining early-life vaccine efficacy [59,60]. Table 2 presents a more detailed comparison of IV vaccines and MLV vaccines. These limitations have motivated the development of next-generation vaccines capable of overcoming maternal antibody interference while eliciting robust, long-lasting immunity.

**Table 1:** Major existing IV and MLV vaccines against BVDV.

Author	Publication Year	First Report/Commercial Launch	Vaccine Name	Type	Target strain(s)	Antigen Protein	Adjuvant	Inactivating Agent	Key Outcomes	Efficacy Measure	Reference
Robert W. Fulton	2020	Early 2000s	CattleMaster^®^ Gold FP 5	IV	BVDV-1a (5960 CP); BVDV-2a (53637 CP)	Viral structural protein	——	——	Strong response to BVDV2a/2c; low proportion of protective antibodies against BVDV1b (20%)	Antibody titers	[47]
Nathan Erickson	2020	Early 2000s	Triangle 5	IV	BVDV-1; BVDV-2	Complete viral particles	——	——	Difficult to elicit an effective primary immune response in the presence of high maternal antibodies (>32 GMT)	Antibody titers	[59]
Maha Raafat Abd El Fadeel	2022	2022	BVD inactivated vaccine with 1% and 2% monolaurin	IV	BVDV-1 (NADL)	Complete viral particles	1% and 2% monolaurin	1% Ascorbic acid	1. Significantly superior efficacy compared with aluminum adjuvants. 2. Immune protection up to 9 months vs. 5 months. 3. Sustained high antibody titers. 4. Excellent safety with no injection-site reactions.	Antibody titers + Duration of protection	[14]
Cunyuan Li	2024	2024	H_2_O_2_ -inactivated BVDV 1 vaccine	IV	BVDV-1 (NADL)	Complete viral particles	1. Complete Freund’s adjuvant (primary) 2. Incomplete Freund’s adjuvant (booster)	H_2_O_2_	1. Immune response ≥ 70 days. 2. Higher IgG and neutralizing antibodies than formaldehyde vaccines. 3. Stimulates humoral and cellular immunity.	Antibody titers + Duration of protection	[3]
Berfin Kadiroğlu	2024	2024	Trivalent Inactivated BVDV vaccine	IV	BVDV-1f, BVDV-1l, BVDV-2b	Complete viral particles	1. Aluminum hydroxide [Al(OH)_3_] 2. Aluminum hydroxide + Saponin 3. Montanide™ ISA 50 4. Montanide™ ISA 206	Binary ethylenimine	1. Oil adjuvants (especially ISA 206) are superior to aluminum. 2. Antibodies appear 21 days post-immunization; persist ≥ 111 days. 3. Neutralizing antibodies against homologous and heterologous strains.	Duration of protection	[50]
Stefano Nardelli	2021	2021	Bivalent MLV	MLV	BVDV-1, BVDV-2	Viral structural protein	——	None	Good neutralizing antibodies against BVDV-1 and BVDV-2; incomplete fetal cross-protection against HoBi-like Pestivirus	Antibody titers	[54]
Robert W. Fulton	2020	Early 2000s	Pyramid 5^®^	MLV	BVDV-1a (Singer CP); BVDV-2a (5912 CP)	Viral structural protein	——	None	High antibody levels against BVDV-1a, 1b, 2a; 73% of calves achieved protective titers (≥128) for BVDV-1b	Antibody titers	[47]
Robert W. Fulton	2020	Mid-2000s	Arsenal^®^ 4	MLV	BVDV-1 (CL 760 NCP)	Viral structural protein	——	None	Strong response to BVDV-1b (84% ≥ 128); weak to BVDV-2a (no BVDV-2 component)	Antibody titers	
Robert W. Fulton	2020	Late 2000s	BowShield Gold^®^ FP 5	MLV	BVDV-1a (NADL CP); BVDV-2a (53637 CP)	Viral structural protein	——	None	Weakest antibody response for BVDV-1a/1b; only 4–5% of calves achieved protective titers for BVDV-1b	Antibody titers	
Robert W. Fulton	2020	Mid-2000s	Vista^®^ 5 SQ	MLV	BVDV-1a (Singer CP); BVDV-2a (125A CP)	Viral structural protein	——	None	Moderate response for BVDV-1a/1b; 37% reached protective antibody titers for BVDV-1b	Antibody titers	
Robert W. Fulton	2020	Early 2010s	Breed Back^®^10 (Express^®^ FP 10)	MLV	BVDV-1a (Singer CP); BVDV-2a (296 CP)	Viral structural protein	——	None	Highest antibody levels for BVDV-1a/1b/2a; 86% reached protective titers for BVDV-1b	Antibody titers	
Małgorzata D. Klimowicz-Bodys	2021	Late 2000s	Mucosiffa^®^	MLV	BVDV-1a (C24V)	Attenuated live BVDV particles	Non-adjuvant	None	1. Single dose protection up to 12 months. 2. Rapid induction of neutralizing and ELISA antibodies. 3. High safety; safe for fetuses. 4. Antibody levels meet protective standards, preventing disease and persistent infection.	Antibody titers + Duration of protection	[42]

Note: “——” indicates information is not provided by the literature.

**Table 2:** Comparison of IV and MLV vaccines against BVDV.

Comparative Dimension	IV Vaccine		MLV Vaccine	
	Overview	Example	Overview	Example
Antigens	Viruses inactivated by chemical or physical means		Artificially attenuated virus	
Immune response	Primarily stimulates humoral immunity, with a relatively weak cellular immune response		Simultaneously stimulates robust humoral and cellular immune responses, mimicking the natural infection process	
Efficacy	Limited efficacy, with good protection against homologous strains but insufficient cross-protection against heterologous strains	CattleMaster^®^ Gold FP 5	Typically more potent and comprehensive, demonstrating significant efficacy against both homologous and heterologous strains	Breed Back^®^10
Duration of immunity	Shorter, Requires more frequent booster shots to maintain protective antibody levels	hydrogen peroxide (H_2_O_2_) inactivated BVDV type 1 vaccine	Long-lasting (typically lasting 6–12 months or longer), capable of establishing immune memory	Mucosiffa^®^
Safety	Greater safety, No risk of infection or virulence rebound	Triangle 5	Risks include virulence rebound, immunosuppression, and adverse effects on pregnant animals	BowShield Gold^®^ FP 5
Field performance	With flexible administration, it can be urgently inoculated at any stage, including epidemic outbreaks and pregnancy periods	the BVD inactivated vaccine with 1% and 2% monolaurin	Vaccination timing should be carefully planned (it is generally recommended to complete vaccination before breeding)	A Bivalent Modified Live Vaccine

## 5. Current Limitations in BVDV Control

### 5.1. BVDV Genetic Diversity and Vaccine Protection

Despite widespread vaccination, traditional BVDV vaccines continue to face significant limitations, often resulting in suboptimal immune protection. One of the primary challenges results from the extensive genetic and antigenic diversity of BVDV. Although numerous subtypes exist, most commercially available vaccines are formulated using only BVDV-1a or BVDV-2a strains. This narrow representation can create gaps in protective coverage, as substantial antigenic variation exists even within the same subtype. Therefore, while vaccination can effectively protect against homologous or closely related strains, cross-protection against genetically distant subtypes, such as 1i, 2b, or 2c, may be insufficient [61].

Epidemiologically, this manifests as breakthrough infections caused by subtypes not included in the vaccine, even in fully vaccinated populations, thereby undermining overall disease control [61]. Moreover, differential vaccine efficacy across subtypes may provide certain viral variants with a competitive advantage, driving selective viral evolution under immune pressure. For example, surveillance in Germany revealed that after the implementation of mandatory control programs and vaccination campaigns targeting BVDV-1a and BVDV-1b, these subtypes declined or disappeared, while BVDV-1d gradually emerged as the dominant subtype. This shift indicates the influence of vaccine-driven selective pressure on viral population dynamics. Additionally, live vaccine strains such as Oregon C24V and KE-9 can persist in calf tissues following maternal inoculation, potentially complicating surveillance efforts and influencing viral ecology [62]. Similar patterns have been observed in other regions. In Brazil, outbreaks of respiratory disease caused by BVDV-2b occurred in herds vaccinated with formulations containing BVDV-1a and BVDV-2a, suggesting that certain subtypes may exhibit a competitive advantage under current vaccine-induced immune pressure [63]. Collectively, the high genetic diversity of BVDV, antigenic mismatches between vaccines and circulating strains, and variability in host immune responses contribute to the continued presence of PI animals within vaccinated herds. These PI animals act as continuous sources of viral shedding, significantly compromising the effectiveness of eradication programs and, in some cases, leading to apparent vaccine failure [13]. The genetic diversity of BVDV may render vaccine-induced neutralizing antibodies incapable of effectively recognizing and neutralizing heterologous strains, resulting in insufficient cross-protection levels. This immune escape, triggered by genotypic differences, manifests directly as vaccinated animals developing clinical infections and viremia despite immune responses, with infected pregnant animals producing PI calves. This clearly demonstrates that antigenic mismatch can cause clinical protection failure. Furthermore, the intrahost viral quasispecies distribution and continuous evolution further widen this genotypic gap [53].

The impact of vaccination on viral population structure and epidemiology highlights the need to consider subtype diversity not only in terms of relative abundance but also in relation to their pathogenic potential. Effective BVDV control strategies must therefore account for the dynamic interplay between vaccine coverage, viral genetic variation, and host immune responses to optimize herd protection and reduce the emergence of dominant, vaccine-escape subtypes.

### 5.2. Vaccination Effectiveness

The effectiveness of traditional BVDV vaccines in pregnant cows is highly dependent on the timing of vaccination, which is critical for protecting the fetus and preventing the establishment of persistent infection. BVDV is capable of vertical transmission across the placenta, and if infection occurs during early gestation, particularly between days 42 and 125, the fetal immune system is not yet fully developed to recognize and eliminate the virus. This immaturity results in the birth of PI calves, which serve as long-term viral shedders and become primary reservoirs for infection within the herd. Vaccination is designed to induce high levels of neutralizing antibodies in the dam, thereby blocking transplacental viral transmission. Completing the full vaccination schedule prior to the critical gestation window and achieving protective antibody titers (e.g., neutralizing antibody titer ≥ 128) substantially reduces the risk of fetal infection. Conversely, delayed vaccination, inadequate antibody responses, or immunosuppressive conditions during pregnancy, such as stress, transport, or concurrent illness, can compromise vaccine efficacy and increase the likelihood of producing PI offspring [7].

A further challenge in evaluating vaccine effectiveness arises from the lack of standardized immunogenicity testing. Virus neutralization (VN) assays, commonly used to assess antibody responses, vary widely between laboratories due to differences in viral strains, serological cross-reactivity, and assay protocols [64]. Discrepancies in sample incubation times, kit selection, and testing conditions for different sample types further exacerbate inconsistencies. These methodological variations hinder reliable comparison of vaccine performance, complicate scientific evaluation of immunogenicity, and limit the ability to formulate evidence-based vaccination strategies [65].

Addressing these limitations requires the development of next-generation vaccines capable of providing broader and more robust protection. Integrating advanced technologies such as high-throughput sequencing and AI can enable real-time monitoring of viral genetic variation, predictive modeling of antigenic epitopes, and rational design of multivalent vaccines. Such approaches will support the precise tailoring of vaccines to circulating BVDV strains, ultimately enhancing fetal protection, reducing PI prevalence, and strengthening overall herd immunity.

## 6. Future Directions

### 6.1. Novel Vaccines

#### 6.1.1. mRNA Vaccines

mRNA vaccines represent an innovative platform in vaccine development, leveraging the principle that mRNA sequences encoding pathogen-specific antigens are encapsulated in lipid nanoparticles (LNPs) and delivered into the host cytoplasm. Once inside, the host’s translational machinery synthesizes the encoded antigenic proteins, eliciting robust humoral and cellular immune responses and establishing long-term immunological memory [66]. Compared with traditional vaccines, mRNA technology offers rapid adaptability and precise antigen design, enabling the swift synthesis of mRNA sequences encoding antigens such as the E2 glycoprotein tailored to circulating BVDV subtypes (e.g., 1d, 2b, or emerging variants) based on real-time epidemiological surveillance [67]. This flexibility allows mRNA vaccines to overcome the limitations of traditional vaccines caused by subtype diversity and antigenic variability, mitigating immune escape and improving coverage against emerging viral strains. The platform has been widely applied to rapidly evolving pathogens, notably in the development of COVID-19 vaccines, demonstrating its potential for a timely response to viral evolution [68].

Recent studies have explored the application of mRNA technology to BVDV. In initial validation studies, two BVDV-1 mRNA vaccines employing cap-dependent and cap-independent (IRES-mediated) translation mechanisms were developed and evaluated in mice, guinea pigs, and goats. Both vaccines elicited high levels of neutralizing antibodies, with the cap-dependent formulation showing superior immunogenicity in mice and guinea pigs. In goats, the antibody titers induced by the mRNA vaccine (≥9 by -log_2_) met protective thresholds comparable to commercial formaldehyde-inactivated vaccines, without significant adverse effects, demonstrating the feasibility and safety of this approach for BVDV vaccination [66].

Building on these foundational studies, subsequent research focused on expanding antigenic coverage and enhancing expression efficiency. A trivalent mRNA vaccine was developed encoding structural domains I-II of the E2 glycoprotein from BVDV-1a, BVDV-1b, and BVDV-2. Antigen expression stability and immunogenicity were further optimized by fusing the E2 sequences with bovine IgG1 Fc fragments (bFc) and STABILON (hStab) sequences. This optimized vaccine elicited high, durable neutralizing antibody responses in mice, guinea pigs, and lactating goats, providing cross-protection across all three genotypes, with overall immunogenicity comparable to commercial vaccines [69].

In summary, mRNA vaccine technology for BVDV has demonstrated substantial progress, combining high efficacy, safety, and multigenotypic protection. Initial studies validated immunogenicity and safety, while subsequent optimization enhanced antigen stability and broadened immune coverage. The successful development of a multivalent mRNA vaccine against BVDV-1a, 1b, and 2 highlights the potential of this platform to provide rapid, durable, and broadly protective immunity, offering a promising pathway for next-generation BVDV vaccines.

#### 6.1.2. Subunit Vaccines

Subunit vaccines offer a promising strategy to reduce viral circulation in the field and improve overall productivity in dairy and beef operations [70,71,72]. Compared with traditional vaccines, subunit formulations provide an excellent safety profile because they do not contain live pathogens, eliminating the risk of infection associated with MLVs while overcoming the relatively low immunogenicity of IVs [73,74].

One of the key advantages of subunit vaccines is their ability to induce cross-protection against both BVDV-1 and BVDV-2. Chowdhury et al. developed a subunit vaccine using BoHV-1 as a vector to express a fusion protein of BVDV-2 E2 and Erns-GM-CSF. Although the vaccine primarily targets the BVDV-2 E2 antigen, high sequence homology between BVDV-1 and BVDV-2 E2 enables conserved epitopes to be recognized by the immune system. Calves immunized with this vaccine generated BVDV-2-specific neutralizing antibodies while also eliciting robust cross-reactive cellular immune responses, demonstrated by IFN-γ secretion and T-cell proliferation against both BVDV-1 and BVDV-2. This cross-reactive memory was rapidly recalled upon BVDV-2 challenge, enhancing protection against heterologous BVDV-1 strains [75].

Recent advancements have expanded subunit vaccine design to multiple antigenic targets. Montbrau et al. evaluated a commercially available subunit vaccine (DIVENCE) composed of recombinant E2 glycoproteins from BVDV-1 and BVDV-2, which elicited high, long-lasting neutralizing antibody titers and demonstrated broad-spectrum cross-protection [76]. Another study developed a subunit vaccine based on the highly conserved capsid C protein, which not only stimulated strong humoral and cellular immunity but also preserved intestinal mucosal integrity by reducing epithelial apoptosis, increasing goblet cell numbers, and enhancing tight junction protein expression, highlighting a dual protective mechanism [77].

Antigen-targeting strategies further improve subunit vaccine efficacy. For instance, fusing E2 with a single-chain antibody targeting antigen-presenting cells (APCH-E2) elicited stronger and more durable neutralizing antibody responses in both guinea pigs and cattle, outperforming conventional inactivated vaccines in field trials [78]. To address BVDV’s high genetic diversity, multivalent subunit vaccines covering five prevalent subtypes (BVDV-1a to 1e) have been developed, offering broader immune coverage [79]. Optimization of antigens and adjuvants also enhances protection; Wang et al. constructed an E2 fusion protein combined with MF59 and CpG-ODN co-adjuvants, demonstrating superior reduction in viremia and tissue damage compared with single-antigen vaccines [80].

In summary, subunit vaccines represent a highly adaptable platform for BVDV control. By focusing on conserved epitopes, targeting multiple structural proteins, and employing antigen-targeting or multivalent designs, these vaccines can elicit broad and long-lasting humoral and cellular immunity. Such precision antigen engineering allows subunit vaccines to overcome the cross-protection limitations of traditional vaccines, offering a pathway toward safer, more effective, and broadly protective BVDV vaccination strategies [81].

#### 6.1.3. Other Novel Vaccines

To overcome the persistent challenges of BVDV and the limitations of traditional vaccines, including safety concerns, restricted cross-protection, and logistical constraints, researchers have developed a variety of next-generation vaccine platforms using modern biotechnological approaches [82]. Among these, multi-epitope vaccines, recombinant vaccines, and DNA vaccines represent three major avenues of exploration, each showing considerable promise.

Multi-epitope vaccines offer the advantages of safety, broad-spectrum immunity, and the simultaneous induction of both humoral and cellular responses [83,84]. Utilizing reverse vaccinology, Wei et al. designed two broad-spectrum multi-epitope vaccine candidates, BVDV-M1 and BVDV-M2, targeting the E0 and E2 envelope glycoproteins. Computational analyses including molecular docking and kinetic simulations demonstrated strong binding affinity to bovine Toll-like receptors (TLR2/TLR4). Immune simulations predicted that these vaccines could elicit high levels of IgG and IgM antibodies, as well as cytokines such as IFN-γ and IL-2. Codon optimization allowed efficient expression in *Escherichia coli*, providing a robust candidate for a broad-spectrum vaccine capable of covering multiple viral subtypes [85].

Recombinant vaccines have also been developed to enhance ease of administration and mucosal immunity. One notable example is an oral recombinant *Lactobacillus casei* vaccine expressing the BVDV E2 protein fused to the cholera toxin B subunit (ctxB). Here, E2 serves as the primary immunogen, while ctxB functions as a mucosal adjuvant to improve antigen delivery and enhance mucosal immune responses [86]. Experimental studies demonstrated that the vaccine tolerated gastrointestinal conditions effectively, activated dendritic cells, promoted Tfh cell differentiation, and induced robust production of both mucosal sIgA and systemic IgG antibodies in mouse models. Moreover, it stimulated a mixed Th1/Th2/Th17 cellular immune response, conferring comprehensive immune protection [87].

DNA vaccines combine stability, ease of production, and the ability to induce both humoral and cellular immunity, while avoiding the vertical transmission risks associated with MLV vaccines [88]. A recent study developed a DNA vaccine by fusing the BVDV-1 E2 glycoprotein gene with the mouse lysosome-associated membrane protein 1 (mLAMP1) molecular adjuvant. Two constructs were tested with the E2 gene inserted at different mLAMP1 regions; the construct in the hinge region exhibited higher in vitro expression. Subsequent animal studies demonstrated strong induction of neutralizing antibodies and E2-specific IFN-γ responses, highlighting the vaccine’s ability to concurrently stimulate humoral and cellular immunity while maintaining safety and ease of production.

In summary, each of these novel vaccine platforms addresses key limitations of traditional BVDV vaccines. Multi-epitope vaccines employ rational design to achieve broad-spectrum humoral and cellular immunity across multiple subtypes. Oral recombinant *Lactobacillus* vaccines facilitate convenient administration and robust mucosal and systemic immunity. DNA vaccines, particularly when combined with molecular adjuvants, elicit potent neutralizing antibody and cellular responses while ensuring safety and production efficiency. Collectively, these approaches establish a strong theoretical and technical foundation for the development of next-generation BVDV vaccines that are safe, effective, and easily deployable (Table 3).

### 6.2. New Technologies

The rapid advancement of bioinformatics and high-throughput technologies, including AI and next-generation sequencing (NGS), is transforming modern vaccine development by greatly improving the precision, speed, and efficiency of antigen discovery and vaccine design [90]. These technologies enable the systematic identification of immunologically advantageous epitopes, the rational design of multi-epitope antigens, and the optimization of structural stability and immunogenicity. Despite their transformative potential, these approaches have not yet been widely applied to BVDV vaccine development. To illustrate their utility, lessons can be drawn from applications in tuberculosis vaccine research.

#### 6.2.1. Sequencing Technology

Sequencing technologies, particularly high-throughput platforms (e.g., NGS), provide a critical data foundation for rational vaccine design. By comparing viral protein sequences from databases such as UniProt and the Immune Epitope Database (IEDB), conserved immune epitopes that are widely recognized across diverse MHC alleles can be accurately identified, supporting the development of broadly protective vaccines [91].

The integration of sequencing technologies with AI has significantly accelerated vaccine discovery for complex pathogens, including Mycobacterium tuberculosis. Using reverse vaccinology, AI-driven computational tools can systematically screen pathogen pan-genomes to identify novel antigen candidates, such as PE26, PPE65, and EsxL, that would be challenging to discover using conventional methods. Whole-genome and transcriptome sequencing provide the molecular data necessary to identify immunodominant epitopes, simulate host–pathogen interactions, and optimize antigen design for enhanced efficacy and safety [92].

Beyond genomics, complementary high-throughput technologies expand the scope of vaccine-relevant data. Proteomic sequencing maps protein expression and post-translational modifications, revealing functional effectors of pathogen biology [93]. Metabolomic profiling captures dynamic changes in small-molecule metabolites, reflecting cellular and pathogen functional states. Spatial transcriptomics integrates gene expression with tissue context, linking molecular activity to anatomical localization [94]. The integration of these multi-omics approaches will enable detailed mapping of virus–host interactions, uncovering spatiotemporal networks of pathogenic mechanisms, and supporting the design of multi-targeted intervention strategies against BVDV [95].

For BVDV specifically, sequencing technology allows direct, sensitive identification of complete or partial viral genomes from clinical or environmental samples. This enables rapid detection of newly emerging or low-prevalence strains that traditional methods may miss, alongside precise genetic characterization. By constructing dynamic molecular epidemiological maps, researchers can track the spatial and temporal distribution and evolutionary trajectories of circulating BVDV strains. Such surveillance informs the selection of vaccine strains that closely match circulating lineages, addressing the problem of antigenic mismatch caused by BVDV’s high genetic diversity. Ultimately, establishing a molecular surveillance network integrating advanced sequencing and bioinformatic analyses is essential for transitioning BVDV vaccines from static formulations to dynamic, precision-guided, and forward-looking interventions, an approach critical for improving global BVDV prevention and control.

#### 6.2.2. Artificial Intelligence

AI is increasingly transforming vaccine development by enabling precise, data-driven design strategies. AI applications in vaccinology can be broadly categorized into three areas. Machine learning utilizes large-scale datasets and statistical models to predict key antigen properties, including antigenicity, solubility, and allergenicity [90]. Deep learning, a subset of machine learning, uses multi-layer neural networks to address complex biological tasks such as protein structure prediction (e.g., AlphaFold), sequence optimization (e.g., ProteinGenerator), and structural design (e.g., RFdiffusion), thereby facilitating the rational creation of novel protein structures and epitope alignments [96]. Reinforcement learning further refines vaccine design by iteratively optimizing specific elements, such as epitope linkers or adjuvant fusion sites, to enhance both stability and immunogenicity [92]. Although AI has not yet been applied to BVDV vaccine design, its utility is exemplified in tuberculosis vaccine research. One study employed comparative epitopeomics to screen potent epitopes from eight major *Mycobacterium tuberculosis* antigens, subsequently constructing a stable chimeric antigen, MtbEpi-17, using deep learning tools (ProteinGenerator, RFdiffusion, and AlphaFold3). Computational simulations indicated high binding affinity to Toll-like receptors and strong activation of both innate and adaptive immune responses, highlighting AI’s potential to overcome traditional vaccine design challenges, including poor antigen stability and suboptimal immunogenicity [96].

For BVDV, AI could predict immunogenicity, cross-protection potential, and antigenic compatibility across diverse viral subtypes. By integrating viral genomic data with host genetic backgrounds and immune characteristics, AI models can help design precision vaccination strategies tailored to specific herds. This approach has the potential to create next-generation BVDV vaccines capable of targeting multiple genotypes while maintaining high safety and efficacy, thereby overcoming the limitations posed by the virus’s extensive genetic and antigenic diversity.

### 6.3. Comprehensive Prevention and Control

While vaccination is essential for controlling BVDV, it cannot achieve eradication alone. Effective control requires the concurrent management of PI animals, which serve as primary viral reservoirs. Even highly efficacious vaccines are constrained by BVDV’s antigenic variability, incomplete cross-protection between subtypes, and continuous viral evolution under immune pressure. If PI animals remain in a herd, they can perpetuate viral transmission despite vaccination, making it difficult to interrupt infection chains and potentially facilitating the emergence and spread of novel viral subtypes [9]. Therefore, a comprehensive control strategy combining vaccination, identification and removal of PI animals, biosecurity measures, and ongoing molecular surveillance is critical for effective and sustainable BVDV management.

Therefore, the sustainable control of BVDV requires the establishment of an intelligent, integrated prevention and control system that combines vaccination, surveillance, diagnostics, and targeted culling programs [97]. This system should be grounded in continuous and systematic epidemiological monitoring, utilizing high-throughput sequencing technologies to genotype circulating viral strains and track their evolutionary dynamics. Such molecular surveillance enables real-time assessment of the match between vaccine strains and field isolates, providing precise guidance for vaccine selection and timely updates. In the diagnostic phase, AI-assisted tools, including image analysis and predictive data models, can integrate clinical signs, production performance metrics, and laboratory test results to improve the early detection of PI animals and subclinical infections [98]. Once PI animals are identified, strict culling protocols must be implemented to remove the primary source of viral transmission. Concurrently, vaccination strategies should be dynamically optimized based on surveillance and sequencing data, prioritizing the protection of breeding females to prevent the birth of new PI calves while establishing herd-level immunity to reduce viral spread [78]. Ultimately, by integrating multi-source data, including pathogen genomes, host immune profiles, environmental factors, and management practices, through AI-driven modeling, predictive decision-support systems can be developed. These systems provide a robust and scientifically guided framework for the sustainable control and potential regional eradication of BVDV [99].

## 7. Conclusions

BVDV causes persistent infection and severe clinical disease through multiple mechanisms, including suppression of host innate immunity, induction of immune tolerance, and metabolic reprogramming. PI animals play a central role in maintaining virus circulation, posing significant economic burdens on the global cattle industry. Traditional vaccine strategies, including IV and MLV vaccines, remain widely used but face several limitations: insufficient antigenic match with diverse circulating subtypes, interference from maternally derived antibodies, and potential risks of MLV vaccination in pregnant animals. Novel vaccine platforms, such as mRNA vaccines, subunit vaccines, multi-epitope vaccines, DNA vaccines, and recombinant vaccines, are still in the experimental stage with no commercial products yet available. However, they offer notable advantages, including high safety, rapid and precise design, and the ability to elicit broad cross-protective immunity, positioning them as promising candidates for future BVDV prevention and control.

Currently, the control of BVDV continues to face several significant challenges. First, there is considerable geographic variability in the distribution of BVDV subtypes, which likely arises not only from the virus’s ongoing mutation and recombination but also from immune-selective pressure exerted by vaccination programs. This dynamic has contributed to the emergence and maintenance of numerous antigenically distinct subtypes. In regions where specific vaccine strains are frequently used, subtypes that differ antigenically may gain a selective advantage, potentially leading to the persistence of mutant strains. The precise mechanisms driving this subtype diversity, however, remain to be fully elucidated. The resulting abundance of subtypes complicates the accurate detection of circulating strains and makes it difficult to assess their prevalence, further exacerbating challenges in BVDV surveillance and control.

Another unresolved issue concerns the mechanisms underlying the increased susceptibility of BVDV-infected animals, particularly PI individuals, to secondary infections. Current evidence suggests that increased susceptibility may be linked not solely to viral persistence but also to dysregulation of the host interferon system [8]. It remains to be determined whether BVDV infection induces overexpression of interferon antagonists, leading to excessive interferon activation, selective immunosuppression, and, consequently, increased vulnerability to opportunistic pathogens. The specific pathways exploited by secondary pathogens in this disease are also not yet well understood.

Looking forward, future BVDV vaccine development should prioritize precise antigenic epitope prediction and intelligent multivalent vaccine design by integrating advanced technologies such as artificial intelligence and high-throughput sequencing. Given the extensive diversity of BVDV subtypes and the regional variability of circulating strains, systematic molecular surveillance should be strengthened to monitor prevalence and guide vaccine strain selection. Simultaneously, efforts should focus on the development of multi-epitope vaccines capable of targeting multiple BVDV subtypes, thereby improving the alignment between vaccines and locally circulating strains. Vaccine design should also employ novel adjuvants to enhance immunogenicity and, where appropriate, consider combination vaccines that target multiple pathogens. Such multivalent formulations could simplify vaccination schedules, broaden protection, and reduce the number of administrations required.

Overall, the sustainable control of BVDV will require a multidimensional approach that integrates effective vaccination, comprehensive detection and culling of PI animals, and stringent biosecurity measures. By addressing the challenges posed by viral subtype complexity, covert transmission mechanisms, and incomplete coverage of current prevention strategies, this integrated framework offers the best prospect for long-term, effective control of BVDV.

## Figures and Tables

**Table 3:** Major novel vaccines currently at the laboratory or preclinical stage.

Author	Publication Year	First Report/Development Stage	Vaccine Name	Type	Target Strain(s)	Antigen	Key Outcomes	Reference
Jing Huang	2024	2024 (preclinical)	Cap-dependent mRNA vaccine	mRNA vaccine	BVDV-1 (NADL)	E2 glycoprotein	Induced neutralizing antibody titers ≥ 9 (−log_2_); capped mRNA reached a peak titer of 13.7 at day 35, significantly higher than the uncapped vaccine (10.1)	[66]
Jing Huang	2024	2024 (preclinical)	Cap-independent mRNA vaccine	mRNA vaccine	BVDV-1 (NADL)	E2 glycoprotein		
Shi Xu	2025	2025 (preclinical)	bFc_BVDV_3E2_ARVLP_hStab mRNA Vaccine	mRNA vaccine	BVDV-1a (NADL); BVDV-1b (JS2201); BVDV-2 (C201602)	E2 glycoprotein	Induced neutralizing antibodies, but with lower magnitude and durability compared with the cap-dependent formulation	[69]
Carlos Montbrau	2025	2025 (preclinical)	DIVENCE	Subunit vaccine	BVDV-1; BVDV-2	Recombinant E2 glycoproteins (BVDV-1 and BVDV-2)	Induces potent, durable, and broad-spectrum neutralizing antibody responses against BVDV-1 and BVDV-2; Possesses reliable DIVA characteristics	[76]
Feifei Liu	2025	2025 (preclinical)	BVDV C Protein Subunit Vaccine	Subunit vaccine	BVDV-1q (HNL-1)	Capsid (C) protein	Demonstrated feasibility of the C protein as a vaccine antigen; however, neutralizing antibody titers require further improvement	[77]
Demian Bellido	2021	2021 (preclinical)	Vedevax BLOCK^®^	Subunit vaccine	BVDV-1a; BVDV-1; BVDV-2a	APCH-E2 fusion protein	Induced strong and long-lasting neutralizing antibody responses, offering high protective efficacy and cost-effectiveness.	[78]
Verónica Avello	2024	2024 (preclinical)	A recombinant subunit vaccine	Subunit vaccine	BVDV-1a, 1b, 1c, 1d, 1e	E2 glycoprotein	Elicited robust humoral and cellular immune responses, with a pronounced Th1 bias; neutralizing antibody responses against BVDV-1a were comparable to commercial vaccines.	[79]
Shenghua Wang	2020	2020 (preclinical)	Recombinant Ems-E2 protein vaccine	Subunit vaccine	BVDV-1 (NADL)	Recombinant E2 fusion protein	Induced strong neutralizing antibodies and T-cell immunity (Th1 and CTL responses), significantly reducing viremia and tissue damage following challenge.	[80]
Min Wei	2024	2024 (in silico/preclinical)	BVDV-M1	Multi-epitope vaccine	BVDV-1a, 1b, 1c, 1k, 1m, 1n, 1q BVDV-2a	E0 (Erns) envelope glycoprotein	Predicted to induce robust humoral and cellular immune responses, supporting broad protection across prevalent BVDV subtypes.	[85]
Min Wei	2024	2024 (in silico/preclinical)	BVDV-M2	Multi-epitope vaccine	BVDV-1a, 1b, 1c, 1k, 1m, 1n, 1q BVDV-2a	E2 envelope glycoprotein		
Shuo Jia	2020	2020 (preclinical)	pPG-E2-ctxB/Lc W56	Recombinant *Lactobacillus* oral live vector vaccine	BVDV-1 (ZD-2018)	E2 envelope glycoprotein	Induced potent mucosal, humoral, and cellular immune responses and effectively cleared BVDV infection in experimental models.	[87]
Yusuke Sakai	2024	2024 (preclinical)	pVax-mLAMP1-E2 (H)	DNA vaccine	BVDV-1 (Nose)	E2 envelope glycoprotein	Induced strong neutralizing antibody responses, with enhanced efficacy following intradermal administration.	[89]

## Data Availability

No new data were created or analyzed in this study. Data sharing is not applicable to this article.
